# Bioprospecting potential and secondary metabolite profile of a novel sediment-derived fungus *Penicillium* sp. ArCSPf from continental slope of Eastern Arabian Sea

**DOI:** 10.1080/21501203.2019.1572034

**Published:** 2019-01-31

**Authors:** Arakkaveettil Kabeer Farha, Abdulla Mohamed Hatha

**Affiliations:** Department of Marine Biology Microbiology and Biochemistry, School of Marine Sciences, Cochin University of Science and Technology (CUSAT), Kochi, India

**Keywords:** *Penicillium*, marine fungi, anticancer, antibacterial, LCMS, secondary metabolites, continental slope, Arabian Sea

## Abstract

Marine fungi, one of the major decomposers of marine environment, is found to produce potential enzymes and novel biomolecules. The present study explored bioprospecting potentials such as antimicrobial, anticancer and enzymatic activities of marine sediment-derived fungi isolated from continental slope of Eastern Arabian Sea. Morphology and ITS sequencing identified the fungus as *Penicillium* sp. ArCSPf. The fungal strain exhibited amylase, gelatinase, phytase, lipase and pectinase activity. The active fraction obtained from the ethyl acetate extract column fractionation (F2) of fungus showed antibacterial activity against both methicillin-resistant *Staphylococcus aureus* (MRSA) and *Bacillus cereus*. Minimum inhibitory concentrations of F2 were 125** **μg/mL for MRSA and 62.5** **μg/mL for *B. cereus*. The active fraction showed a significant anticancer activity (**IC_50_ = 22.79** µg/mL) against MCF-7 breast cancer cells. The secondary metabolite (Z)-Octadec-9-enamide (oleamide, *m*/*z* 282.27 (M + H^+^)] was identified in the LC-MS/MS analysis of active fraction F2 in positive ionisation mode. To the best of our knowledge, this is the first report on exploring the bioprospecting potential of a sediment-derived fungus from continental slope of eastern Arabian Sea for the production of therapeutically active compounds.

## Introduction

The bioprospecting of marine-derived fungi, an underexplored resource of biodiversity, provides potent biomolecules with a wide range of therapeutic applications. So far, more than 1000 potent biomolecules with anticancer or antibacterial activities from marine fungi have been reported (Gomes et al. 2015). However, none of them entered into the pharmaceutical global market till date due to the lack of information regarding their taxonomy and mode of interaction with biological targets (Deshmukh et al. 2017). Therefore, secondary metabolites from marine fungi warrant a great consideration as a future drug source for discovering novel therapeutics. Besides its ability to produce natural products, fungi represent a rich source of enzymes with relevant biotechnological and industrial applications.

Natural products synthesised by fungi could be an alternative option in the drug discovery strategy because of its non-toxic nature and easy availability from nature (Nigam and Singh 2014). The bioactive potential of a strain is correlated with their chemical profile of secondary metabolites, and screening and characterisation of these metabolites are found to be the key concepts of metabolomics (Roy and Banerjee 2017). Liquid chromatography-mass spectrometry (LC-MS) analysis is frequently employed to screen potential bioactive secondary metabolites of microbial strains (Lee et al. 2011).

*Penicillium* genus is widely distributed in marine environment and has received considerable attention due to the presence of secondary metabolites. To date, majority of bioactive secondary metabolites are reported from genus *Penicillium* (Capon et al. 2007; Silber et al. 2016). In the present study, the enzyme potential, antibacterial and anticancer activities of *Penicillium* sp. from marine sediments collected from continental slope of eastern Arabian Sea were evaluated. Chemoprofiling of the metabolites in fungal extract was done using LC-MS/MS analysis.

## Materials and methods

### General experimental

Culture media and agar were procured from HiMedia while solvents were purchased from Merck. Dulbecco’s Modified Eagle Medium (DMEM), Fetal Bovine Serum (FBS) and antibiotic-antimycotic solution were purchased from Invitrogen. MCF-7 breast cancer cell line was provided by National Centre for Aquatic and Animal Health (NCAAH), Kochi, India.

### Fungal isolation and molecular identification

Marine sediment was collected from continental slope of eastern Arabian Sea (Kochi transect, 500** **m depth/09.56 N; 75.32 E). The fungus was isolated from sediments by serial dilution method followed by plating on Sabouraud Dextrose Agar (SDA). The pure fungal strain was maintained on SDA plates for further studies. Different media such as Czapek Dox agar, Chloramphenicol-Rose Bengal agar, Yeast Extract Peptone Dextrose (YEPD) agar and SDA were used for the identification of colony morphology of fungus.

The genomic DNA isolated from the fungus was subjected to amplification of Internal Transcribed Spacer (ITS) of the fungal ribosomal DNA by Polymerase chain reaction (PCR) using the following primers: forward-UL18F: 5´-TGTACACACCGCCCGTC-3´ and reverse-UL28R: 5´-ATCGCCAGTTCTGCTTAC3´. The PCR cycling condition was as follows: initial denaturation at 95°C for 10 min followed by 35 cycles of denaturation at 95°C for 30 s, annealing at 60°C for 40 s and elongation at 72°C for 1 min. The purified PCR product was subjected to partial DNA sequencing by Sanger’s method. The gene sequence of fungus was compared with the data available in NCBI using BLAST. The phylogenetic analysis was performed and phylogenetic tree was constructed using Molecular Evolutionary Genetics Analysis (MEGA) software version 4.1 and sequence was deposited in GenBank.

### Enzyme studies

Spot inoculation method was used for the detection of extracellular hydrolytic enzymes produced by isolated fungal strain. Basal Mineral Agar (BSA) medium [KH_2_PO_4_ – 0.1%, (NH_4_)_2_SO_4_ – 0.5%, MgSO_4_ 7H_2_O – 0.01%, NaCl – 0.01%, and agar 2.0%] supplemented with specific substrates such as 1% soluble starch, 1% Tween 80 (v/v), 1% carboxymethyl cellulose, sodium phytate, methylene blue, skim milk, chitin, pectin and gelatin were used for the detection of amylase, lipase, cellulase, phytase, ligninase, caseinase, chitinase and gelatinase enzyme activity, respectively. The fungal strain was inoculated on plates supplemented with the respective substrates and incubated at 28°C for 2 days. Amylase activity was detected by the addition of 1% iodine solution while cellulase activity was detected by adding 0.1% Congo red solution. Gelatinase activity was detected by addition of 1% mercuric chloride. The formation of clear zone around the fungal strain was noted (Farha et al. 2018).

### Preparation of fungal extract

Pure culture of fungus was inoculated into 10 L Czapek Dox medium and incubated at room temperature for 21 days with shaking at 120 r.p.m. At the end of incubation, the mycelia were homogenised with a blender. The whole medium including mycelia was subjected to maceration by adding equal volume of ethyl acetate (1:1) based on cold percolation extraction procedure. The flasks were kept for 5 days with intermittent shaking and on the 6th day, the mixture was filtered to remove debris. The ethyl acetate extract was evaporated using rotary evaporator to get a dried and concentrated extract. The ethyl acetate extract showing bioactivities was then chromatographed using solvents of different polarity. The order of solvents used in column chromatography was n-Hexane (F1), n-Hexane/ethyl acetate (1:1) (F2), ethyl acetate (F3), ethyl acetate/methanol (1:1) (F4) and methanol (F5). A total of five fractions were collected and screened for their anticancer/antimicrobial activity by using disc diffusion method. The active fraction, F2, shows that bioactivity was dissolved in DMSO at a concentration of 1 mg/mL and used for further studies.

### Antibacterial assay by Disc diffusion method

A total of four clinical pathogenic strains *Klebsiella pneumoniae, Pseudomonas aeruginosa, Escherichia coli* and methicillin-resistant *Staphylococcus aureus* (MRSA) were kindly provided by Microbiology wing, Medical Trust Hospital, Kochi, India. *Bacillus cereus* used in the study was obtained from our laboratory collection. The strains were plated on Muller Hinton (MH) agar. Sterile paper disc impregnated with 10** **μL of active fraction F2 of fungal culture was placed on MH agar plate. The plate was incubated for 24 h at 37°C. Zone of inhibition was measured.

### Minimum inhibitory concentration (MIC)

The minimum inhibitory concentration of active fraction F2 was determined using 96-well microplate method (Elshikh et al. 2016). The active fraction F2 dissolved in DMSO was diluted with MH broth by double dilution method and 100 µL of MH broth was dispensed in each well. To each well, 5 μL of bacterial suspension (10^8^ CFU/mL) was inoculated. The plate was incubated for 24 h at 37°C. Resazurin (0.015%) was added to each wells (30 µL/well) and further incubated for 2 h for the observation of colour change. At the end of incubation, wells retaining the blue colour of resazurin were noted. The concentration of extract in that column was taken as the MIC value. The experiment was conducted in triplicate.

### Minimum bactericidal concentration (MBC)

The minimum bactericidal concentration (MBC) was determined by plating directly the content of wells with concentrations higher than the MIC value. The MBC value was determined when there was no colony growth from the directly plated contents of the wells (Elshikh et al. 2016).

### Anti-breast cancer activity

The anticancer activity of the active fraction F2 was determined by following the method of (Kabeer et al. 2017). MCF-7 cells were cultured in DMEM supplemented with 10% FBS and antibiotic-antimycotic solution and maintained in an incubator at 37°C and 5% CO_2_. Briefly, 5 × 10^3^ cells/100** **μL/well were seeded in 96-well plates and incubated overnight. The cells were treated with various concentrations of cative fraction F2 and further incubated for 24 h. Hundred microliter of 3-(4,5-Dimethylthiazol-2-yl)-2,5-diphenyl-tetrazolium bromide (MTT) (5 mg/mL) was added to each well and incubated at 37°C for 2 h in the dark. DMSO (100** **μL) was added and further incubated at 37°C for 4 h. The absorbance at 570 nm was recorded using microplate reader (Thermo fisher Varioskan LUX).

### LC/MS analysis

The active fraction F2 was first dissolved in methanol and then re-dissolved in water. The sample was analysed on an Agilent 6540 Q-TOF MS coupled to Agilent 1290 uHPLC system. Source Parameters were as follows: Gas temp: 320°C; Gas flow: 6 L/min; Vcap: 4000 V; Fragmentor voltage: 180 V; Skimmer voltage: 65 V; Ion polarity: Positive. Acquisition parameters: MS (*m*/*z* range): 50–2000; MS scan rate: 3 spectra/s; Collision energy: 15** **V. Extend C18 (2.1 × 50 mm, 1.8** **μm) column was used for the separation with solvents 0.1% formic acid in water and 0.1% formic acid in acetonitrile (CAN) and a flow rate of 0.2 mL/min. The identification of compounds was done by comparing the mass spectra with standard spectra available in Metlin library and available literature data.

## Results

### Isolation of marine fungi from sediments

**Figure 1.**
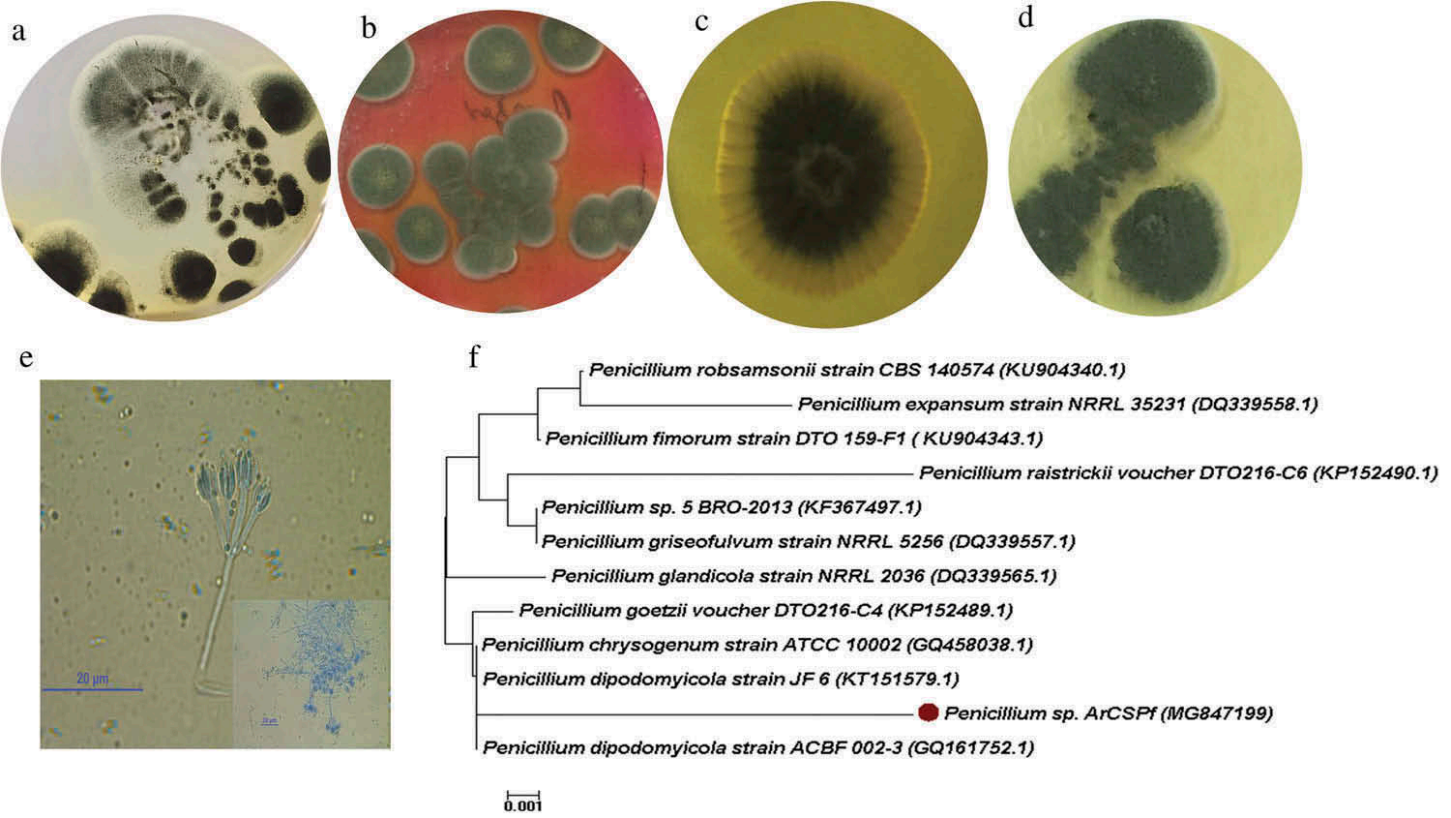
Morphology of *Penicillium* sp. ArCSPf on different agar medium. (a) Czapek Dox agar. (b) Chloramphenicol-Rose Bengal agar. (c) Yeast Extract Peptone (YEPD) agar. (d) Sabouraud dextrose agar. (e) Microscopic image of *Penicillium sp*. ArCSPf. (f) Phylogenetic tree based on ITS1-5.8S-ITS2 sequences showing relationship between *Penicillium* sp. ArCSPf and related *Penicillium* species.

From the continental shelf sediment sample collected from eastern Arabia Sea, 12 fungi were isolated. Out of the 12 fungi, one was selected for further studies. The colony morphology of fungus on different culture media such as Czapek Dox agar, Chloramphenicol-Rose Bengal agar, YEPD agar and SDA is shown in Figure 1(a–d). The fungal strain showed production of soluble pigments (yellow coloured zone around the colony) when grown in YEPD agar. White border appearance with dark green conidia is observed when cultivated in all the media. Microscopic analysis of fungus stained with lactophenol cotton blue showed a paintbrush like appearance with chains of single cell conidia on the conidiophores. Both macroscopic and microscopic studies revealed that the fungus belonged to *Penicillium* sp. (Figure 1(e)).

### Molecular characterisation of fungi

The ITS1–5.8S–ITS2 regions were amplified and subjected to partial sequencing for the identification of fungus. Sequence similarity search using NCBI BLAST (Basic Local Alignment Search Tool) returned several closely related sequences. A phylogenetic tree was constructed, using the neighbour-joining method (Figure 1(f)). The sequence data showed 99% homology with *Penicillium dipodomyicola* strain. The sequence was submitted in GenBank database as *Penicillium* sp. ArCSPf (Accession No. MG847199).

### Hydrolytic enzyme activity

The hydrolytic enzyme production potential of the fungal strain *Penicillium* sp. ArCSPf revealed that they are able to secrete exoenzymes such as lipase, gelatinase, amylase, phytase and pectinase into the medium. The extend of hydrolysis is measured in terms of diameter of inhibition zone which revealed very good lipolytic activity of *Penicillium* sp. ArCSPf (Table 1). This fungal strain showed the following order of enzyme activity: lipase > phaytase > amylase > gelatinase > pectinase. The *Penicillium* sp. ArCSP was unable to produce caseinase, cellulose and chitinase enzymes.

**Table 1.** Hydrolytic enzyme activity of *Penicillium* sp. ArCSPf (*n* = 3, mean ± SD).

Enzyme activity	Zone of clearance (mm)
Lipase	5 ± 0.3
Gelatinase	3 ± 0.4
Amylase	4 ± 0.2
Phytase	4 ± 0.3
Pectinase	3
Caseinase	0
Cellulase	0
Chitinase	0

### Antibacterial activity

**Figure 2.**
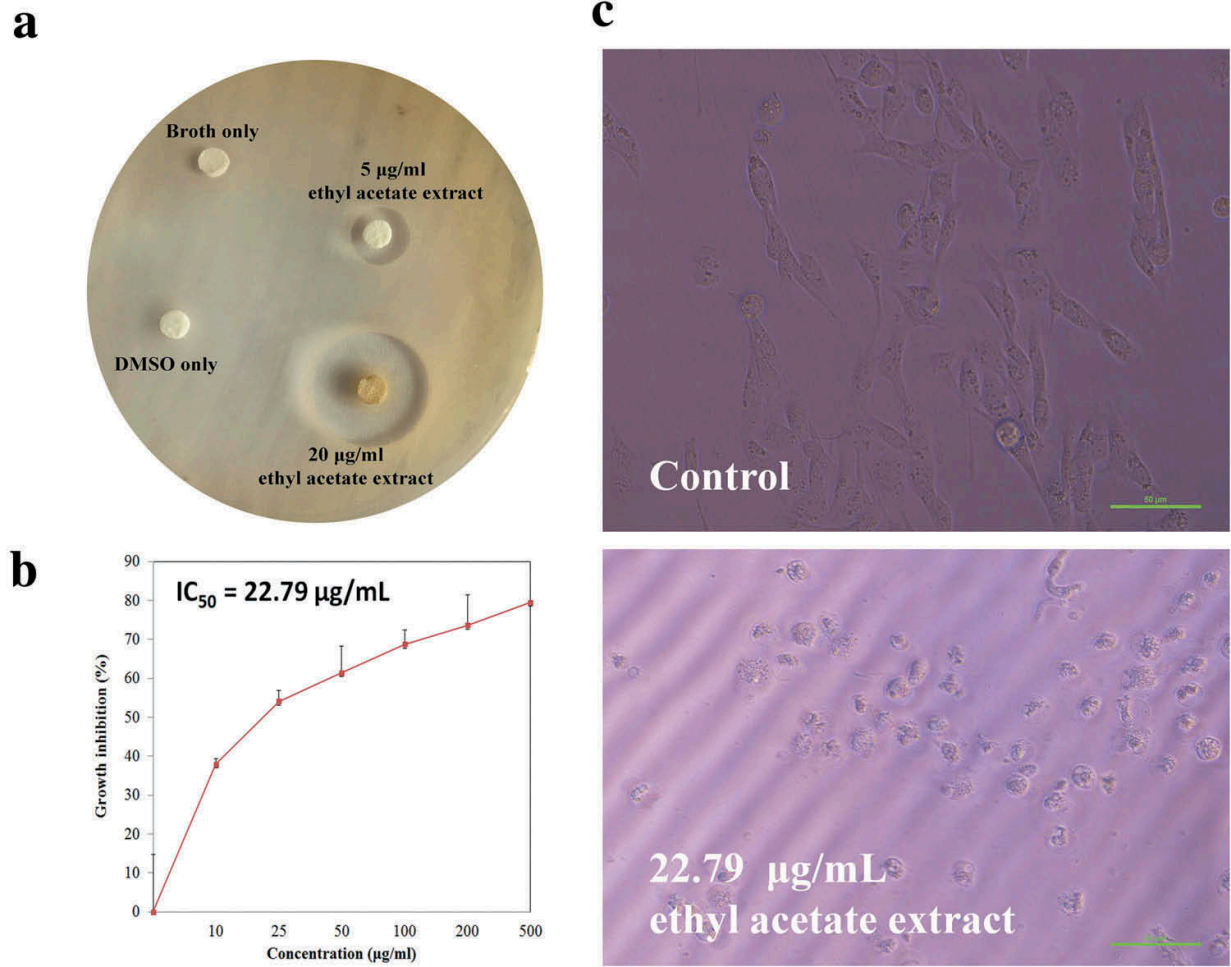
Bioprospecting potential of *Penicillium* sp. ArCSPf. (a) Antibacterial activity. (b) *In-vitro* cytotoxic analysis by MTT assay. (c) Morphology of MCF 7 cells before and after treatment with F2 fraction of *Penicillium* sp. ArCSPf.

The n-Hexane/ethyl acetate (1:1) fraction (F2) was tested for the antibacterial activity against Gram-positive bacterial pathogens namely methicillin-resistant *S*. *aureus* (MRSA) and *Bacillus cereus* and Gram-negative bacteria such as *E*. *coli, K. pneumoniae* and *P*. *aeruginosa* by disc diffusion method. The antibacterial activity is measured in terms of diameter of inhibition zones. Fraction F2 showed promising antibacterial activity against MRSA and *B. cereus* with diameter of inhibition zones 21 mm and 19 mm, respectively. Fraction F2 also inhibited the growth of MRSA in both the concentrations (5 and 20** **μg/mL). On the other hand, fraction F2 did not show any zone of inhibition against *E. coli, K. pneumoniae* and *P. aeruginosa* indicating that the antibacterial activity is specific to Gram-positive forms (Figure 2(a)).

### MIC and MBC

Table 2 represents the MIC and MBCs values of fraction F2 against MRSA and *B. cereus*. The antibacterial activity of fraction F2 seems to be directly proportional to its concentration. When the concentration of F2 increased from 3.91** **μg/mL to 250** **μg/mL, the inhibitory effect also increased proportionally. Results also revealed that higher concentration of fraction F2 is required to inhibit the growth of MRSA. The MIC values of active fraction F2 of *Penicillium* sp. ArCSPf were 125** **μg/mL and 62.5** **μg/mL for MRSA and *B. cereus*, respectively. Similar trend is also reflected in MBC values. The MBC values for active fraction F2 against MRSA and *B. cereus* were 250** **μg/mL and 125** **μg/mL, respectively.

**Table 2.** Minimal inhibitory concentration (MIC) and minimal bactericidal concentration (MBC) of active fraction F2 of *Penicillium* sp. ArCSPf against bacterial strains.

Tested microorganisms	MIC (µg/mL)	MBC (µg/mL)
Methicillin-resistant *Staphylococcus aureus* (MRSA)	125	250
*Bacillus cereus*	62.5	125

### Anti-breast cancer activity

The evaluation of cytotoxicity of active fraction F2 of *Penicillium* sp. ArCSPf was carried out using breast cancer MCF-7 cells. MCF-7 cells were treated with varying concentrations (0–500 µg/mL) of fraction F2 for 24 h and observed for viability. A significant reduction in the viability of MCF-7 cells was observed when the concentration of F2 increased. IC_50_ value of F2 was found to be 22.79 µg/mL (Figure 2(b)). At higher concentration (500 µg/mL), active fraction F2 was able to inhibit more than 75% of MCF-7 cells. It is also noted that exposure to F2 resulted change in the morphology of MCF-7 cells and ultimate loss of its viability. The dead cells became round in shape and detached from the surface of flask (Figure 2(c)). These results indicated that F2 inhibited *in vitro* proliferation of MCF-7 cancer cells in a dose dependent manner.

### LC-MS/MS analysis

**Figure 3.**
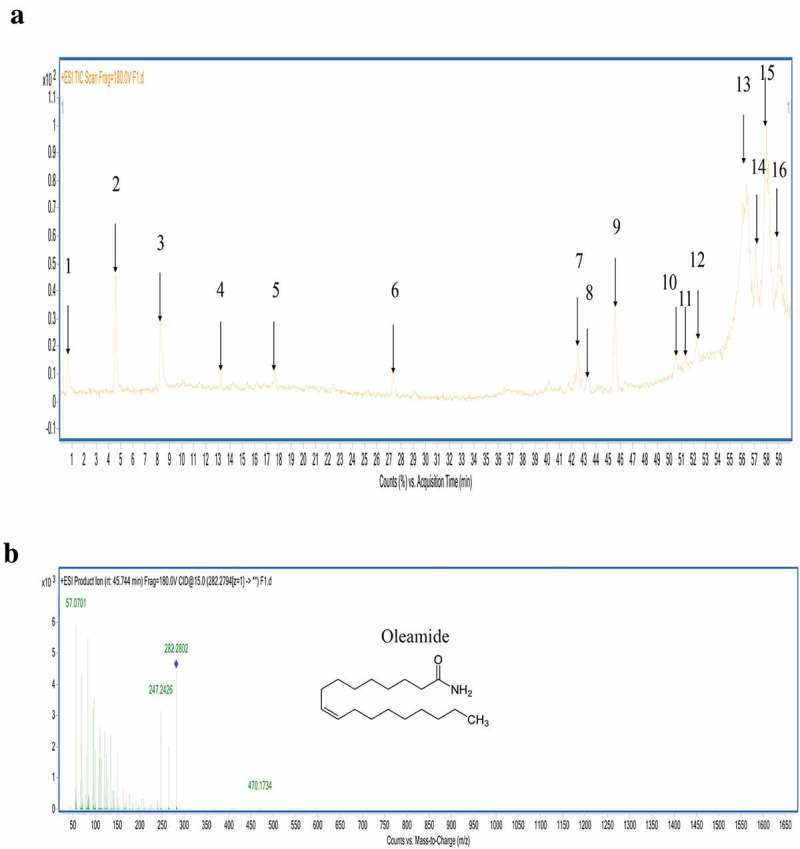
(a) The total ion chromatograms (TIC) of the active fraction of *Penicillium* sp. ArCSPf. (b) MS–MS spectrum of oleamide.

LC-MS/MS analysis in positive ionisation mode showed the presence of 16 peaks in the total ion chromatograms (TIC) of the active fraction of *Penicillium* sp. ArCSPf ethyl acetate extract (Figure 3(a)). LC-MS/MS analysis identified the presence of 10 dominant compounds in the active fraction. Chemical structure of the compound was identified by comparing the MS/MS spectrum of compounds with that of known compounds in Metlin library. Table 3 shows the fragmentation pattern of compounds in the active fraction F2. Nine compounds remained unidentified, as the spectral data did not match with any compound in the Metlin library. (Z)-Octadec-9-enamide (oleamide) was the identified compound in the F2 fraction. LC-MS/MS analysis in positive mode ionisation showed the presence of peak of *m*/*z* 282.27 (M + H^+^) indicating the presence of oleamide in the active fraction F2. MS/MS fragmentation pattern showed the presence of specific peaks at 57.0701, 282.2802, and 247.2426, which confirmed the presence of oleamide (Zaher et al. 2014) (Figure 3(b)).

**Table 3.** Fragmentation pattern (MS/MS) of identified peaks in the active fraction F2 of *Penicillium* sp. ArCSPf by LC-MS/MS analysis.

Peak	Retention time	(M + H^+^) *m/z*	Molecular weight (MW)	Fragmentation (MS/MS)
1	1.158	136.9310	135	56.9468,118.9207,136.93
2	4.49	231.07	230	80.0328,159.0540, 231.0743
3	8.14	475.32	474	86.0957,214.8596,278.8715,358.8174,432.7737,475.3254
4	13.02	255.15	254	57.0694, 255.1558, 215.8574
5	17.41	359.18	358	55.6599,164.0453, 282.8414, 359.1828
6	27.27	437.19	436	45.2602,120.9644,240.9113,390.8164,437.1936
7	42.37	507.27	506	72.6696,174.4637,237.2185,311.1241,507.2720
8	42.92	475.32	474	86.0957,214.8596,278.8715,358.8174,432.7737,475.3254
**9**	**45.744**	**282.27**	**281**	**57.0701,282.2802,247.2426** **Oleamide**
10	50.334	449.36	448	124.0760, 337.2342,449.3606, 494.8037
11	51.50	449.36	448	337.2341,449.3607,684.4007
12	52.05	449.36	448	160.0683, 260.5178,337.2341,449.3607,651.1357
13	55.438	449.36	448	156.07, 224.3387, 337.2351,489.0472,606.5470,449.3600
14	56.96	413.26	412	123.116, 337.2351,489.0472
15	58.24	449.36	448	255.8639, 337.234,449.36
16	58.76	449.36	448	150.023, 244.8639, 337.234,449.36,598.82

## Discussion

Fungi inhabiting in the deep marine sediments are least studied group of microbes with promising therapeutically active compounds. These bioactive compounds are found to be very effective against bacterial infection and cancer. Recent studies reported the diversity of fungi in Arabian Sea (Soumya et al. 2013). Despite their abundance in the marine environment, bioprospecting potential of marine fungi from continental slope of eastern Arabian Sea is not much explored. As part of our ongoing studies to identify cytotoxic compounds from marine fungi, the present study examined marine fungi by culture-dependent method and evaluated the bioprospecting potential and metabolite profile of the isolated fungal strain.

In the present study, fungal strain isolated from marine-sediment sample was identified as *Penicillium dipodomyicola* by ITS rDNA sequencing and designated as *Penicillium* sp. ArCSPf. *Penicillium* sp. was found to be one of the most common and dominant genera in marine environment because of its ability to tolerate high salt concentration, low temperature, pressure variation etc. Studies on filamentous fungal diversity from continental slope of Arabian Sea showed that approximately 43% of the fungal isolates belonged to the genera *Penicillium* (Soumya et al. 2013).

Marine-derived fungi have been reported to produce enzymes with potential biotechnological applications due its novel physiological characteristics, such as high salt tolerance, thermostability, barophilicity, and cold-activity (Bonugli-Santos et al. 2015). However, there are no published reports on the bioactivity of marine sediment borne fungi from eastern Arabian Sea and our report seems to be the first in this regard. *Penicillium* sp. is found to be good producers of extracellular enzymes that can degrade various complex polysaccharides, lipids and proteins (Ranjana and Randhir Babu 2016). The present study showed the capability of *Penicillium* sp. ArCSPf strain to produce various extracellular hydrolytic enzymes such as lipase, gelatinase, amylase, phytase and pectinase. Among extracellular enzyme activities, lipase activity is found to be superior. The results are in accordance with the previous study, which reported that *Penicillium* sp. S36 from Arabian Sea exhibited significant lipase activity (Smitha et al. 2014). It is probable that these extracellular hydrolytic enzymes may involve in the degradation of complex organic matter in the marine sediment and play key role in the marine carbon cycle.

In the current study, active fraction F2 was only effective against Gram-positive bacteria and has shown remarkable inhibition of Gram-positive pathogens such as MRSA and *B. cereus*. MIC values 125 and 62.5** **μg/mL of active fraction F2 against MRSA and *B. cereus*, respectively, were also quite impressive. This specific activity against Gram-positive pathogens will come handy when it comes to the development of narrow spectrum antibacterial agents. These findings corroborated with the early studies that reported that marine-derived *Penicillium* sp. exhibited significant antimicrobial activity (Devi et al. 2012; Noor Ifatul et al. 2016). These results indicate that *Penicillium* sp. ArCSPf strain is highly promising. F2 fraction also displayed significant anticancer activity against MCF 7 cancer cells through the inhibition of proliferation of cancer cells. The cell death was confirmed through the identification of morphological changes such as cell shrinkage, loss of membrane integrity, rounded and detached cells by microscopic analysis. The bioactivity of F2 fraction might be due to the presence of chemical compounds present in the extract. *Penicillium* sp. is a prolific producer of secondary metabolites with anticancer and antibacterial potential (Gao et al. 2010; Xu et al. 2015; Jouda et al. 2016). Several compounds isolated from *P. dipodomyicola* have been reported as anticancer/antibacterial drugs including Speradine B, griseofulvin, epigriseofulvin and isogriseofulvin and Peniphenones A-D (Porsani et al. 2013; Li et al. 2014; Wang et al. 2015).

The chemical characteristics of active fraction F2 were determined by LC-MS/MS analysis and based on the spectral data 9-octadecenamide (Z) (Olemide) was identified as one of the compounds in the fraction with the molecular formula C**₁₈**H**₃₅**NO and molecular weight 281. The presence of oleamide, a bioactive fatty acid ester, has been reported in plants and fungi (Kima et al. 2010). Oleamide is reported to produce antibacterial (Devi and Muthu 2014) and other pharmacological effects. Zaher et al. (2015) identified oleamide as a component in the crude extract of *Botryodiplodia theobromae*, which exhibits antimicrobial activity. Oleamide was identified from the fungi *Fusarium fujikuroi* (Loureiro dosReis et al. 2018) and *Colletotricum gloeosporioides* (Premjanu and Jaynthy 2015). Biosurfactants from halophilic *Halomonas* sp. BS4 contains 9-octadecenamide (Z) that has shown antibacterial and anticancer activities (Donio et al. 2013). While the present study provides the idea of utilising marine *Penicillium* fungi as an ideal source of potent biomolecules, more studies are warranted to identify the unknown compounds in the active fraction. Due to lack of information, the other peaks were not matched to previous studies of known compounds and hence not identified. This may be due to the presence of unknown compounds that were not previously reported from *Penicillium* species.

To the best of our knowledge, this is the first report on exploring the bioprospecting potential of a sediment-derived fungus from continental slope eastern Arabian Sea for the production of therapeutically active compounds and extracellular hydrolytic enzymes. The active fraction F2 of the fungus contained oleamide and exhibited strong anticancer and antibacterial activities. The present study suggested that *Penicillium* sp. ArCSPf is a promising candidate, which could be used as a source for the discovery of biomolecules. Further studies on the various enzymes and identification of unknown compounds will be warranted.
